# Editorial: Voices from the frontline: the lived experiences of healthcare professionals in the workplace

**DOI:** 10.3389/fsoc.2025.1619110

**Published:** 2025-06-06

**Authors:** Maria Berghs

**Affiliations:** Leicester School of Allied Health Sciences, De Montfort University, Leicester, United Kingdom

**Keywords:** healthcare, professionals, skills, discrimination, investment, experiences

The COVID-19 pandemic has illustrated how critical it is to invest in healthcare systems and develop professionals as assets to our local and global public health resources. It also ensured more attention was paid to understanding the physical and mental health risks of working in the healthcare sector to ensure proper protections. Most existing research on the experiences of healthcare workers focused on frontline workers during the height of the pandemic (Søvold et al., 2021). Yet, even prior to the pandemic, impacts of: globalization of care, staff shortages, advanced technologies, epidemiological transitions and need for new skills and competencies for 21st century were understood (Pruitt and Epping-Jordan, 2005). Healthcare professionals also face a variety of stressors including increasing workloads, surveillance, patient violence and lack of political support. Those risks have only been exacerbated by a financial crisis, increasingly hostile workplace environments, and even warfare now targeting healthcare professionals (Ioannidis, 2024). Furthermore, differing types of discriminations and racism against healthcare staff, from both patients and colleagues, are prevalent in healthcare systems worldwide (Okeahialam et al., 2025).

This edited Research Topic highlights the lived experiences of healthcare professionals in the workplace. It comprises of nine articles examining workplace challenges in China, Jordan, Italy, Sweden, the United States, Canada and Saudi Arabia. The articles cover topics such as: the lack of investments in GPs and personal care attendants; deteriorating workplace conditions; violence and discriminations; risks of burnout in surgeons; and how to ensure interventions to help better prepare healthcare professionals to manage their emotions.

Genova and Lombardini's commentary picks up on the idea of public health investments in General Practitioners (GPs) or family doctors as cornerstone of primary care. They examine data from 21 European countries looking at whether the number of GPs per 100,000 inhabitants increased or decreased between 1995 and 2014. Most European healthcare systems increased the number of GPs coherently with WHO recommendations. They suggest that a country like Italy, which has not invested in family doctors and thus in the primary care sector in last two decades, would have been less equipped to manage pandemics.

The COVID-19 pandemic also illustrated the importance of personal care attendants for older adults and persons with disabilities, especially if primary care is no longer accessible. Wendel et al. described how critical personal care attendants were during the COVID-19 pandemic in the United States but how their services are not always given recognition. They found personal care attendants do not have equitable pay or professional recognition but they do see themselves as having intrinsic rewards in their work and social support. Yet, a lack of professionalism and investment will compromise future care and quality. Care services are increasingly moving from the clinic to the home. Ensuring greater professionalism of this caring workforce will become a necessity and while not explicit in their work, they point to new forms of discriminations, such as ableism and ageism in healthcare (Simmonds and Berghs, 2024), in careism, a discrimination which views embodied and other forms of care-giving as less important and as less entitled to professional status.

While training, recognition and renumeration are important, so are the conditions in which healthcare professionals work. Xiao et al.'s cross-sectional study investigated the impact of workplace violence against healthcare workers in China, finding that 58.2% of healthcare professionals reported experiencing at least one experience of workplace violence in the past year with emotional abuse being the most commonly reported. Alnaeem et al. also examined workplace violence against nurses in Jordan. They found that 59.6% of the nurses reported verbal abuse was common in their workplace. Both illustrate hierarchies in professions, gendered discriminations and that violence is becoming more commonplace globally.

To understand more about the abuse and racism that health care professionals experience, Ahlberg et al. found that ethnic minority staff in Sweden often have to engage in additional emotional labor. To be a professional means to deal with dilemmas such as discussing racism but also to ethically treat racist patients. They explain how staff had strategies to manage such dilemmas but this had a physical and emotional (traumatic) toll on the health and wellbeing of healthcare professionals. They note how a politics of fear now intersects with professional experiences of hostile environment to immigrants or people who they perceive as “other” countering ideas of necessity of global and diverse workforce.

Minorities and their experiences were also the focus of Bizzeth and Beagan's qualitative study in Canada. They explored heath care professionals' experiences with work-related microaggressions and heteronormativity. They coin the innovative idea of “heteroprofessionalism” and argue that the concept of professional carries encoded within it demands that the occupant of that category be—or present as—heterosexual, an unmarked status that can be readily desexualized. They point to a need to rethink what the boundaries are of professional and ethical behaviors in practice, in keeping with new forms of identities and discriminations experienced.

McNeill et al.'s study also examined professional boundaries in risks of burnout among surgeons in Canada. They found a complex picture of inequitable remuneration associated with education, administration, and leadership roles correlated to the Fee-For-Service model, as well as issues of gender inequity and the individualistic culture that develops as surgeons specialize. They noted how surgeons often had answers for these problems. They suggested to reform payment plans, hospital policies and ensure more social and practical support to combat loneliness and inequalities.

More research is now also concentrating on how to strengthen mental health and resilience through interventions. Alotaibi's study investigated the impact of perceived behavioral control, attitudes, subjective norms, and emotion-focused coping on willingness to treat viral-infected patients in Saudi Arabia. They argue for interventions such as training programs focused on infection control measures and patient management strategies, so healthcare professionals can feel more emotionally and practically prepared in future pandemics. Carminati's article too focuses on the role of emotions at work for healthcare professionals and how emotional management is part of the job but does not always lead to emotional intelligence. They argue that the integration of emotional management and emotional intelligence could bridge the gap between these two pivotal abilities and foster better behavioral and mental health.

All of the contributors point to the needs to invest in rethinking healthcare professionalism with novel competencies, such as: emotional and physical self-management; skills for resiliencies; and specialized training against violence, discriminations and racism. This will demand local and global investments in healthcare systems, as well as creation of new jobs and roles to ensure its sustainability.
